# Organization and implementation of mass medical rescue after an earthquake

**DOI:** 10.1186/2054-9369-1-5

**Published:** 2014-04-22

**Authors:** Yan-Ling Zhang

**Affiliations:** Health Department, General Logistics Department, Chinese People’s Liberation Army (PLA), Beijing, 100842 China

**Keywords:** Disaster, Earthquake, Medical rescue, Military medicine, Military operation, Non-war

## Abstract

On May 12, 2008, an 8.0-magnitude earthquake occurred in Wenchuan, Sichuan Province. In this disaster, 69,000 people were killed, 18,000 people were reported missing, and 37,000 people were injured, including more than 10,000 who were seriously injured. Trauma was the most commonly observed type of injury, with fractures accounting for 74% of all injury cases. On April 14, 2010, a 7.1-magnitude earthquake occurred in Yushu of Qinghai Province. In this disaster, 2,698 people were killed, 270 people were reported missing, and 11,000 people were injured, including more than 3,100 who were seriously injured. Fracture injury accounted for 58.4% of all injury cases. After each earthquake, the Chinese Army Medical Services responded promptly, according to the previously established guidelines, and sent out elite forces to the disaster areas, with the objectives of organizing, coordinating and participating in an efficient and evidence-based medical rescue effort. After the Wenchuan earthquake, 397 mobile medical service teams including 7,061 health workers were sent to the disaster areas. A total of 69,000 casualties were treated, and 22,000 surgeries were performed. After the Yushu earthquake, 25 mobile medical service teams involving 2,025 health workers were sent. They performed 1,635 surgeries and created an astounding outcome of “zero deaths” in the aftermath of the earthquake during their treatment of casualties in a high-altitude region. Within a week after each earthquake, the military teams rescued approximately 60% of the total number of rescued casualties and evacuated approximately 80% of the total number of evacuated sick or wounded victims, playing a critical role and making invaluable contributions to earthquake relief. The experience and lessons learned from the rescue efforts of the Chinese military after the two earthquakes have highlighted several key aspects in emergency medical rescue: (1) medical rescue theories must be updated; (2) military-civilian cooperation must be stressed; (3) professional rescue forces must be strengthened; (4) supporting facilities must be improved; and (5) international exchanges and cooperation must be deepened.

China is located at the junction of the world’s two major seismic belts – the circum-Pacific seismic belt and the Eurasian seismic belt – and is one of the world’s most earthquake disaster-ridden countries. Over the past century, a total of 40 earthquakes measuring ≥7 on the Richter scale have occurred worldwide, of which 10 took place in China and killed nearly six hundred thousand people (accounting for 53% of the total number of global deaths due to earthquakes) [1]. In recent years, two catastrophic earthquakes occurred in China, one in Wenchuan of Sichuan Province and the other in Yushu of Qinghai Province, both of which caused heavy casualties. The Chinese military fully participated in the medical rescue after these two major earthquakes, representing a rescue relief force that had the most rapid reaction and best equipment, undertook the most difficult tasks, and rescued the largest number of wounded or sick victims. Within a week after the earthquake, Chinese military forces rescued approximately 60% of the total number of rescued injured patients and transported approximately 80% of the total number of transported sick and wounded victims, playing a critical role and making important contributions to earthquake relief.

## Overview of the involvement of Chinese military forces in medical rescue efforts after the Wenchuan and Yushu earthquakes

### Medical rescue efforts after the Wenchuan earthquake

On May 12, 2008, a catastrophic earthquake measuring 8.0 on the Richter magnitude scale occurred in Wenchuan, Sichuan Province. This disaster was the most devastating earthquake since the establishment of the People’s Republic China (PRC), affecting the widest region and posing the most difficult for relief efforts. This disaster killed 690,000 people, left 180,000 people missing and 370,000 people injured, including more than 10,000 who were seriously injured. Trauma was the most common type of injury, and fracture accounted for 74% of all injured cases. After the earthquake, the Chinese military sent 397 mobile medical service squads consisting of 7,061 medical workers to the disaster area to participate in the emergency medical rescue, and they treated 69,000 injured people and performed 22,000 surgeries [2].

### Medical rescue efforts after the Yushu earthquake

On April 14, 2010, a violent earthquake measuring 7.1 on the Richter magnitude scale occurred in Yushu of Qinghai Province, which lies at an altitude of more than 4000 meters. This disaster killed 2,698 people and left 270 people missing and 11,000 people injured, including more than 3,100 who were seriously injured. Fracture injury accounted for 58.4% of all injury cases. The Chinese military sent 25 mobile medical service squads consisting of 2,025 medical workers to the disaster area to participate in the emergency medical rescue, and they performed 1,635 surgeries, resulting in the miracle of “zero deaths” during the treatment of mass earthquake casualties and sick people after this earthquake in cold and high altitude regions.

## Main practices

### Taking actions according to the law

The Chinese military participated in the disaster relief by complying with the “Emergency Response Law of the PRC” and the “Regulation on the Army’s Participation in Disaster Rescue”. These two regulations clearly specify the major tasks of the Chinese military when participating in disaster relief; the methods of coordination with local government, authority and procedures for the use of military forces; and the need for military-civilian joint command, disaster preparedness during non-disaster times and financial and material support. After both the Wenchuan and Yushu earthquakes, the Chinese Central government set up an earthquake relief headquarters headed by a commander-in-chief who was a leader of the State Council, and the military played a core role in implementing the decisions disseminated from the headquarters. Meanwhile, the Central Military Commission established an army earthquake relief command headed by a leader of the General Staff to coordinate the action of military forces in the disaster area. The military medical services made rescue preparations and took actions according to the law.

### Prompt response

Time is life, and a prompt response is an essential requirement during mass casualty rescue after an earthquake disaster. After the Wenchuan and Yushu earthquakes, the Chinese military immediately launched the emergency response mechanism, arranged medical service teams to rapidly go to the disaster areas by flexible transport methods (by land, water, air or other means), and established a chain of command, treatment, evacuation and support in a relatively short period of time. Within 60 h after the Wenchuan earthquake, the Chinese army sent 87 squads consisting of 2,346 medical workers from nearby regionss, to the disaster area to carry out the medical rescue. These forces accounted for 60% of all medical care workers involved in the rescue (Table 1). Within 30 min after the Yushu earthquake, three medical teams arranged by the Chinese military started off immediately from Xining and Beijing and arrived at Yushu in the same evening. They built “tent hospitals” in the town of Jiegu, the hardest hit area, and won time for the treatment of the wounded within the “golden relief time” [3, 4].

**Table 1:** **Medical service forces and health resources available within 60 hours after Wenchuan earthquake**

Time after earthquake (h)	Types of medical service forces	Number of medical service forces
2	Medical rescue team	1 team consisting of 24 members
12	Medical rescue team and pandemic control team	28 teams, including 846 members
13	Medicines and health facilities	400,000,000 Yuan (RMB)
	Blood storage for war	20,000 bags
48	Medical rescue team and pandemic prevention team	58 teams, including 1,600 members
	Medical facilities for field medical rescue team	More than 60 sets
	Medical technology vehicle for field war	20
	Health resources	43,000,000 Yuan (RMB)
60	Mobile health service forces	2,346 persons (60% of all first-line medical rescue forces)

### Use of elite forces

The use of elite troops and the nearest military forces and the scientific organization of rescue teams are the basic principles followed by the Chinese military medical services to dispatch medical rescue forces. First, the best medical service forces were deployed to the disaster area. After the Wenchuan earthquake, all the mobile medical rescue squads heading to the disaster area came from PLA General Hospital, the affiliated hospitals of the Military Medical University and other medical institutions with high medical and technical levels. More than 70% of the members of these teams possessed master or doctoral degrees, and there were five academicians from the Chinese Academy of Sciences and the Chinese Academy of Engineering. The professional and technical levels of these rescue teams were the highest among the rescue teams involved in disaster rescue operations that have occurred since the founding of PRC. For example, academician Shi-bi Lu, an internationally renowned orthopedic expert from the PLA General Hospital who was already 79 years old and had previously participated in earthquake relief four times, participated in the rescue effort. He and academician Xiang-mei Chen, a nephrologist, contributed greatly to the treatment of the wounded based on their extensive clinical experience, particularly in guiding the prevention and treatment of crush injury syndrome [5]. Second, military medical service forces were mobilized from the place that was the nearest geographically to the disaster area. In the afternoon of the occurrence of the Wenchuan earthquake, medical institutions near the disaster area, such as the General Hospital of Chengdu Military Command, were ordered to send rescue teams to the disaster area. Third, the best medical equipment was sent to the disaster area. In the rescue effort after the Wenchuan earthquake, two module field hospitals, 20 tented field hospitals and more than 200 field ambulances, operation vehicles, X-ray diagnosis vehicles, and remote consultation vehicles were sent to the affected areas together with the mobile medical service teams. Fourth, rescue teams were organized scientifically. Based on the task requirements, different team sizes (large-scale medical teams consisting of approximately 100 members, medium-scale medical teams consisting of approximately 30 members, and small-scale medical teams consisting of approximately 10 members) were created. In the personnel composition of medical teams, specialists in different fields were included. In particular, collocation of gynecology, pediatrics, dermatology, psychology and other professional specialties in the medical rescue teams played an indispensable role. In the Yushu disaster area, 154 infants were born in two field hospitals.

### Efficient coordination

**Figure 1:**
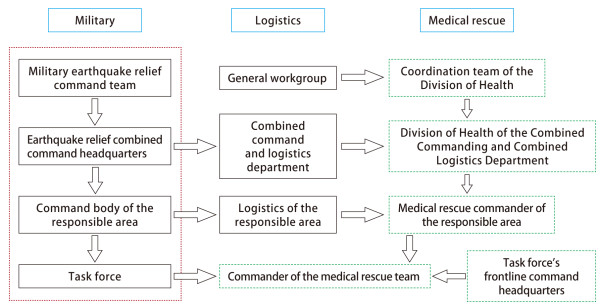
**Command system of the military medical service during the medical rescue in the Wenchuan earthquake area.**

Earthquake disaster medical rescue involves a wide range of forces and therefore requires complex coordination and organization. To improve rescue efficiency and effectiveness, the Chinese military medical services primarily took the following means and measures to organize and coordinate mass casualty rescue. First, the command level of medical service was increased to enhance the authority. The medical service command was included in the entire earthquake disaster rescue command system, and a medical command position was set in the joint medical command headquarters to integrate national, military and local rescue forces. Second, the medical service command level was reasonably set up to enhance timeliness. In the Wenchuan earthquake disaster relief, a four-level medical service command system was implemented, including a strategic medical service, theater medical service, mission area medical service and medical service squad (Figure 1), while a three-level medical service command system was implemented in the Yushu earthquake disaster rescue, including a strategic medical service, theater medical service and medical service squad. Third, collaboration mechanisms were developed to enhance rescue effectiveness. In the Yushu medical relief efforts, a joint conference system, an important-matters consultation system and a regular liaison officer meeting system were established in the disaster areas among the Chinese military medical services, the Chinese national health departments, and the health departments of Qinghai Province and Yushu Tibetan Autonomous Prefecture to collaborate actions, effectively overcome the problem of disorderly and inefficient rescue activities, and ensure orderly and effective rescue operations.

### Scientific organization

In the early stage of the rescue effort, search and rescue was a top priority because of the rapidly increasing number of the wounded. Chinese military medical services combined the medical treatment forces together with the army rescue forces to conduct a simultaneous live search and rescue of the wounded to maximize the possibility of saving critically injured people. In areas with a great number of wounded people, module field hospitals and tent hospitals were created to treat the sick and wounded. After the Wenchuan earthquake, 60 medical service squads were integrated into 19 field hospitals to achieve a comprehensive integration of functional modules to facilitate mass casualty treatment. In areas where the victims were scattered, 30-person medical teams were split into multiple smaller squads containing 3–5 members, and these small squads, together with rescue workers, were dispatched to villages to conduct careful searches so that no casualty was missed. After the wounded were triaged and received emergency treatment, they were evacuated promptly based on the priorities set by the triage to receive definitive treatment as early as possible [5]. In the Wenchuan earthquake rescue relief, ten thousand wounded people were transported to hospitals across the country by air, rail, or road. In the Yushu earthquake relief, 1,640 wounded people were sent by air to hospitals in Xining and Lanzhou, and no deaths occurred during or after transport.

## Experiences and lessons

### Medical rescue theories must be updated

Compared to peacetime health services and wartime medical support, disaster medical relief has its own characteristics; the military background, environmental conditions, nature and function, organization and coordination, health service forces and medical treatment differ significantly (Table 2). Medical service departments and hospitals should provide new medical rescue theories based on their previous practice of disaster relief after earthquakes, landslides, tsunamis and other disasters in different natural environments (plateau, extreme heat or cold and other special circumstances) and develop prevention drugs, specialized equipment, treatment technologies and contingency plans, thereby providing strong theoretical guidance and support for disaster medical relief and improving disaster health management and emergency medical rescue capability [6].

**Table 2:** **Comparison of health services between disaster relief and peace/war time**

Item	Medical support in disaster relief	Peace-time health service	War-time health service
Military background	Emergent state of peace time	Routine state of peace-time	At war
Environment condition	Location of event	Location of training and daily activity	Battle field
Functions	Fighting team, supporting team	Supporting team	Supporting team
Organizing and commanding	Military/local government/military and civil combination/United Nations	Military	Military
Health service forces	Mainly with mobile health service forces	Scaled and local health service forces	Mainly with mobile health service forces
Medical evacuation	On-location first aid, treatment at early stage, specialized treatment	Medical evacuation	Battlefield first aid, emergent treatment, early stage treatment, specialized treatment

### Military-civilian cooperation must be stressed

In medical relief efforts after catastrophic earthquakes, a large number of military and/or civilian rescue institutions from different systems are involved in the rescue work in a local area in a very short period of time. Thus, rescue organization and coordination are very difficult but are important. In medical relief efforts after the Wenchuan earthquake, although headquarters were set up at national, local, and military levels, the problem of military-civilian coordination still existed, and rescue activities were disorderly and inefficient. To effectively overcome this problem, the Chinese military medical services attempted to create a military-civilian cooperation mechanism to coordinate activities at the national, local and on-site levels, enhance information sharing, hold joint meetings, consult major issues, and thereby improve overall efficiency and effectiveness.

### Professional rescue forces must be strengthened

**Figure 2:**
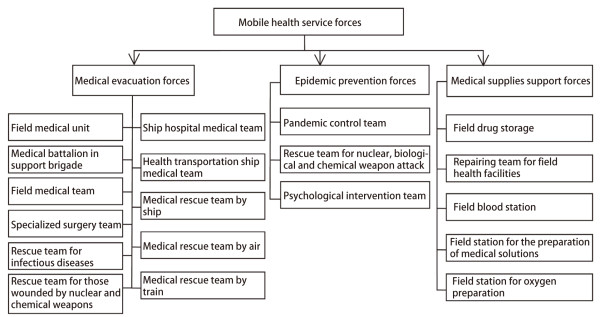
**Construction of the mobile health service forces system.**

Earthquake and other disaster medical relief activities are highly professional and emergent. In peacetime, the Chinese military medical services are integrated in hospitals, military medical universities and other health care institutions. Based on the tasks to be performed, 19 types of mobile emergency medical service teams are created, which can be divided into three categories, i.e., medical treatment, vaccination and protection, and drug support (Figure 2). These teams are equipped with medical equipment that can be rapidly and flexibly expanded under field conditions, and they can develop an earthquake emergency medical rescue plan, conduct targeted training, and carry out a variety of disaster medical relief missions without any pre-disaster practice or material supplies. Previous practices have demonstrated that it is feasible and effective to combine peacetime and wartime practices to strengthen the construction of professional mobile emergency teams. In the future, Chinese military health services should focus on the task of building national professional emergency teams to further strengthen the medical rescue forces.

### Supporting facilities must be improved

In view of the equipment deficiencies and problems encountered in medical rescue after the Wenchuan and Yushu earthquakes, the Chinese military health services should in the future strengthen the construction of supporting facilities in the following four aspects to meet the needs of medical disaster relief support. First, the model of a combination of basic modules, supplementary modules, and single-species modules should be adopted to provide sufficient medical equipment and drugs for medical service teams. For medical rescue after earthquake disasters, in addition to modules for the treatment of fracture injury, head trauma and crush injuries, supplementary modules should also be equipped for psychological management, dermatology, gynecology and pediatrics. Second, large-scale medical treatment and evacuation platforms for medical support forces should be developed to improve mass casualty treatment and evacuation capacity, including trains, field module hospitals or tent hospitals that can be deployed by air, hospital ships, aeromedical transport, including airplanes and helicopters. Third, various types of medical service teams should be equipped with telemedicine systems to allow remote consultation, diagnosis, and surgery to achieve global, full-time, mobile medical treatment and to enhance remote technical support capability. Fourth, national medical rescue teams should be equipped with various types of advanced communication equipment and life support equipment to improve mobility, effective command and field survival capability in harsh environments.

### International exchanges and cooperation must be enhanced

Medical rescue after a catastrophic disaster requires not only military-civilian cooperation but also international cooperation and support [7]. After the Wenchuan earthquake, more than 170 countries and over 30 international organizations provided substantial assistance, including funds, medications, and tents, and the armies and defense ministries of nearly 20 countries provided direct assistance. Natural disasters are challenges faced by all humans. We hope that, under the coordination of the United Nations, the World Health Organization, the International Red Cross, the International Committee of Military Medicine and the Working Group of the Pan-Pacific International Committee of Military Medicine, global military health services can (i) carry out extensive exchanges and cooperation, (ii) create assistance mechanisms that can provide mutual support to rescue forces, (iii) provide advanced technology and equipment, (iv) build platforms for sharing information, and (v) actively conduct joint rescue operations and exercises. Such activities will improve the overall capacity of countries around the world to perform mass medical rescue activities after catastrophic disasters.

## References

[1] Zhang YL (2011). Chinese military health service in non-war time. Med J Chine PLA.

[2] Zhang YL (2009). Ponderation on the strategy of medical rescue after Wenchuan earthquake. Med J Chine PLA.

[3] He ZJ, Ma JX (2005). Time efficiency of first-aid to war wound and trauma. Med J Chine PLA.

[4] He ZJ (2004). Golden ten minutes - a new concept of trauma emergency. Med J Chine PLA.

[5] Lu SB (2008). Importance of triage in medical rescue after the Wenchuan earthquake. Med J Chine PLA.

[6] Sheng ZY (2008). Ponderation on the construction of war or traumatic wound discipline in military hospitals. Med J Chine PLA.

[7] Wang ZG, Guo SS, Zhang LL (2010). International military medicine: frontier issues and implications. Med J Chine PLA.

